# Soybean Lectin Enhances Biofilm Formation by *Bradyrhizobium japonicum* in the Absence of Plants

**DOI:** 10.1155/2009/719367

**Published:** 2009-05-26

**Authors:** Julieta Pérez-Giménez, Elías J. Mongiardini, M. Julia Althabegoiti, Julieta Covelli, J. Ignacio Quelas, Silvina L. López-García, Aníbal R. Lodeiro

**Affiliations:** Instituto de Biotecnología y Biología Molecular, Departamento de Ciencias Biológicas, Facultad de Ciencias Exactas, Universidad Nacional de La Plata-CONICET, Calles 47 y 115 (1900) La Plata, Argentina

## Abstract

Soybean lectin (SBL) purified from soybean seeds by affinity chromatography strongly bound to *Bradyrhizobium japonicum* USDA 110 cell surface. This lectin enhanced biofilm formation by *B. japonicum* in a concentration-dependent manner. Presence of galactose during biofilm formation had different effects in the presence or absence of SBL. Biofilms were completely inhibited in the presence of both SBL and galactose, while in the absence of SBL, galactose was less inhibitory. SBL was very stable, since its agglutinating activity of *B. japonicum* cells as well as of human group A+ erythrocytes was resistant to preincubation for one week at 60°C. Hence, we propose that plant remnants might constitute a source of this lectin, which might remain active in soil and thus favor *B. japonicum* biofilm formation in the interval between soybean crop seasons.

## 1. Introduction

Rhizobia comprise a diverse group of soil bacterial species that share the ability to form N_2_-fixing nodules in legume roots. As part of this group, *B. japonicum* specifically infects and nodulates soybean roots. A great deal of symbiotic specificity relies on a lipochitooligosaccharide molecule synthesized by rhizobia, known as Nod factor. It consists of a backbone of *β*-1,4 linked N-acetyl glucosamines with an N-acyl substituent at the nonreducing end and several decorations in some of its sugar residues. These decorations are responsible for the specific recognition of a given Nod factor by the symbiotic legume species [1]. Another yet uncharacterized specific recognition mechanism is provided by plant lectins [2]. 

The process of early rhizobia-legume interactions that leads to root infection and nodulation has been studied in detail [3]. However, only a minority of rhizobia from the soil are able to occupy the nodules: while a legume growing in natural conditions typically have up to a few hundred nodules in its root, each one occupied by a clone derived from one or a few rhizobial cells, the root system explores a soil volume of several cm^3^, that may contain in the order of 10^7^ rhizobia able to nodulate it [4]. Thus, by focusing on signal exchange, infection, and early nodulation, we probably overlook the fate of 99.99% of the rhizobial population that interacts with the root, but not necessarily produce an effective infection. Turning to the time dimension, we can consider the annual rhizobial cycle as composed by plant infection, nodulation, nodule maturation, senescence, rhizobia release, and persistence in soil. In this view, the early interaction period comprises less than 10% of the whole cycle, while the steps from nodule senescence to rhizobial persistence in soil occupy more than 60% of it. These considerations underscore the importance of the free-living, non-root-associated rhizobial state. In comparison to our knowledge on early infection and N_2_ fixation, few studies were addressed at this out-of-the-root state, in which rhizobia must survive in a very complex and often hostile environment, being exposed to feast-to-famine nutrient fluctuations, cycles from flooding to desiccation, temperature extremes, predation, exposure to UV irradiance when near the soil surface, and inhibition by antibacterial substances like antibiotics released by other members of soil biota. Therefore, we addressed this study at *B. japonicum* biofilm formation in the absence of plants, which seems the preferred state of rhizobia in naked soil, nonsymbiotic rhizospheres, or noninfectable tissue on symbiotic roots.

Biofilms consist in multicellular structures where bacteria are surrounded by extracellular polymers leaving open channels inside the structure, which sets on a surface and acquires typical shapes [5]. Inside biofilms bacteria undergo physiological changes in relation to individual, planktonic cells, leading to special proteomes and metabolic activities [6–8]. The extracellular matrix, mostly composed by exopolysaccharides (EPSs), is believed to play a key role in biofilm endurance [5]. Biofilm formation was first reported in rhizobia by Seneviratne and Jayasinghearachchi in 2003, who observed that *Bradyrhizobium* sp is able to form typical biofilm structures on diverse biotic and abiotic surfaces [9]. Furthermore, roles of EPS [10–12] and Nod factor [13] were observed, as well as the conditioning of biofilm formation by nutrient and osmotic cell status [14].

Although many reports exist on the participation of bacterial lectins (particularly, those taking part in pilus structure) in biofilm formation [15], studies on the participation of plant lectins are lacking. In 1974 Bohlool and Schmidt [16] observed that soybean lectin (SBL) bound specifically to 25 of 28 *B. japonicum* strains, while did not bind to 23 strains from different *Rhizobium* species. Soon these observations were expanded to other rhizobial species and the SBL receptor in the *B. japonicum* surface was located at the EPS [17]. This is in agreement with the general role of lectins in mediating cell-cell contacts through its binding to cell surface polysaccharides. For instance, hemagglutinating activity of SBL by binding erythrocyte surface polysaccharides was known long before a role was assigned to this protein in rhizobia agglutination [18]. Therefore, SBL may contribute to biofilm formation in *B. japonicum* by bridging cells together through their EPS even in the absence of plants, beyond its role on root hair infection [2, 3]. It seems plausible since SBL is released from the plant roots [19] and therefore could be present in the soil surrounding the roots even after their death, provided the protein activity has sufficient stability. In this way, SBL might modify soil environment to facilitate *B. japonicum* biofilm formation in the same site where host plants proliferate or will be established in the next cycle.

However, earlier studies on the participation of SBL in *B. japonicum* adhesion to soybean roots rendered conflicting results: it seemed to have no role during the initial process of rhizobial adhesion, but was able to modify the symbiotic capabilities of rhizobia when these were exposed to small concentrations of SBL during several hours [20, 21]. In other examples, the use of plant or algal lectins was proposed as inhibitor of biofilm formation against dental colonizers [22, 23]. Hence, a direct contribution of SBL to biofilm formation by *B. japonicum* seems not obvious. We addressed this question here by assessing SBL influence on biofilm formation on inert surfaces with a microtiter plate assay [10].

## 2. Materials and Methods

### 2.1. Plants and Bacteria

 Soybean Don Mario 4800 was kindly provided by Alejandro Perticari (IMyZA, INTA-Castelar, Argentina). *B. japonicum* USDA 110 was obtained from USDA, Beltsville, USA, and ΔP22 was kindly provided by Peter Müller, Marburg University, Germany. Both strains were grown and maintained in yeast extract-mannitol [24].

### 2.2. Purification of SBL

 Soybean seeds were ground and sieved through a 0.84 mm mesh. This powder was suspended in N-hexane for 1 hour at −20°C, filtered, and air-dried. Then it was suspended in modified (MFS) N-free Fåhraeus solution [25] for 2 hours at 4°C with stirring, and centrifuged at 10000× g 20 minutes. The supernatant was fractionated with ammonium sulfate between 40 and 70% saturation, and after resuspension and desalting, it was loaded into an *ε*-aminocaproyl-N-acetyl-*β*-D-galactosamine agarose affinity column (Sigma Chemical Co.) at a rate of 3 mL h^−1^ at 4°C [21]. The nonretained fraction of protein material was pooled and the column washed until absorbance at 280 nm reached the blank value. Then, SBL was eluted with 1 M galactose, and finally SBL was pooled, desalted by extensive dialysis against double-distilled water, and lyophilized. All the fractions were conserved at −20°C. Protein concentrations were determined with the Bradford method as described with bovine serum albumin (BSA) as standard [26].

### 2.3. Protein Analysis

 Denaturing polyacrylamide gel electrophoresis (SDS-PAGE) was done as previously described [27] with 5% polyacrylamide in the stacking gel and 12.5% polyacrylamide in the separating gel. For native PAGE, 7.5% polyacrylamide was employed, and SDS and reductants were ommited. Gels were stained either with Coomassie Brilliant Blue or silver [28], as indicated. 

For immunoblot identification, samples were dropped on a polyvinylidene fluoride membrane (immobilon-P Millipore), which was blocked with low fat powder milk and treated with a rabbit anti-SBL antibody obtained from Sigma Chemical Co. For development, an alkaline phosphatase-labeled antirabbit IgG (Sigma Chemical Co.) was employed [21].

### 2.4. Agglutinating Activity

 This was performed with five-day-old yeast-extract mannitol grown *B. japonicum* USDA 110 cells washed and resuspended in PBS (10^10^ cell mL^−1^) or fresh human group A+ erythrocytes suspended in PBS (2% v/v) essentially as described [29, 30]. Agglutinating activity was assessed as the reciprocal of the maximum SBL dilution able to cause cell agglutination.

### 2.5. Biofilm Formation

 Rhizobia were grown as above to an OD_500_ of 1.0. Then, rhizobia were diluted in MFS to an OD_500_ of 0.1. The microtiter plate assay for biofilm quantification was used as described by Fujishige et al. [10]. Briefly, 150 *μ*L of cells or MFS were added to individual wells of a 96-well polystyrene plate. The plates were sealed with sterile parafilm “M” and incubated at 28°C. At different times the medium was removed and the OD_500_ was measured to verify that there was no difference in growth rate among the wells. Then, the biofilms were stained with 0.1% crystal violet for 20 minutes. Dye excess was washed and optical density at 570 nm was recorded. To evaluate the role of SBL on biofilm formation, an aliquot of the indicated concentration of SBL (or the molecule to be assessed) was added at the beginning of the experiment, together with the rhizobia. In no case there were subsequent additions of either molecule to the developing biofilms.

### 2.6. Adhesion to Soybean Roots

 Rhizobia were grown in yeast extract-mannitol at 28°C and 180 rev min^−1^ rotary shaking to an optical density at 500 nm (OD_500_) of 0.5. Then, a method previously described was used [20, 31]. In brief, 10 seedlings per treatment were incubated for 4 hours in MFS with a rhizobial suspension of approximately 10^3^ cells mL^−1^ at 28°C with rotary shaking at 50 rev min^−1^. Viable colony-forming units (CFUs) plate counting at the beginning and at the end of these incubations showed that no loss of viability occurred during incubation. Rootlets with adsorbed rhizobia were washed four times, each by shaking with fresh MFS for 1 minute at 120 rev min^−1^. After washing, the rootlets were distributed on the bottom of petri dishes, and overlaid with molten (45°C) yeast extract-mannitol agar supplemented with cycloheximide and the appropriate antibiotic concentration for selection of the assayed indicator strain. After plate incubation at 28°C, rhizobia remaining adsorbed on the embedded root surfaces developed microcolonies, which were counted along the visible surface of each primary root under a stereomicroscope at 25× magnification. Then we estimated the total number of rhizobial CFU on the whole root surface as described [20, 31]. Total counts of microcolonies on all primary roots, expressed as the percent of the total number of CFU present in the original inoculum, represented the adhesion index, *%A*. Confidence intervals (*P* < .05) were obtained by employing a relationship already described [31], which takes into account the binomial distribution of adhesion index.

## 3. Results

### 3.1. Evaluation of SBL Purification

**Figure 1:**
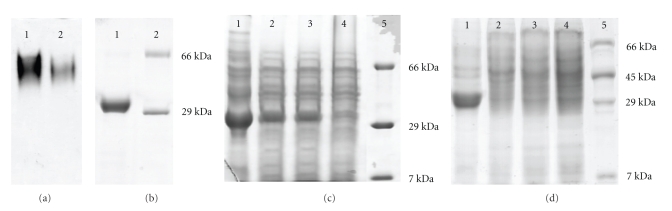
Polyacrylamide gel electrophoresis of SBL samples. (a) Product from affinity chromatography purification procedure in silver-stained nondenaturing PAGE at pH 8 and with a polyacrylamide concentration of 7.5%. *Lane 1*: commercially obtained SBL (Sigma Chemical Co.), *lane 2*: product from affinity purification. (b) The same SBL fraction in silver-stained SDS-PAGE. *Lane 1*: final SBL purification fraction; *lane 2*: molecular weight markers. (c) The same SBL fraction in Coomassie blue-stained SDS-PAGE after incubation with *B. japonicum* USDA 110 cells and centrifugation. Cells were incubated with 10 *μ*g ml^−1^ SBL for 12 hours and centrifuged at 10.000 × g for 20 minutes without further processing (*lane 1*), or after three cycles of resuspension in different solutions, agitation at 4°C 10 minutes and centrifugation at 10.000 × g for 20 minutes. *Lane 2*: resuspension in low salts buffer, containing 3.0 mM KCl, 1.5 mM KH_2_PO_4_, 68.0 mM NaCl, and 9.0 mM NaH_2_PO_4_; *lane 3*: resuspension in 1 M NaCl; *lane 4*: resuspension in PBS containing 1 M galactose; *lane 5*: molecular weight markers. (d) Comparison of the protein profiles from USDA 110 and the mutant ΔP22 after incubation with or without 10 *μ*g ml^−1^ SBL for 12 hours followed by centrifugation at 10.000 × g for 20 minutes. *Lane 1*: USDA 110 with SBL. *Lane 2*: USDA 110 without SBL. *Lane 3*: ΔP22 with SBL. *Lane 4*: ΔP22 without SBL. *Lane 5*: Molecular weigh markers.

 The process of SBL purification was described earlier and is based on affinity chromatography employing the sugar hapten N-acetyl *β*-D-galactosamine as ligand. This process allowed obtaining pure SBL from a complex soybean seed extract, with a high yield. This process rendered a single band in SDS-PAGE electrophoresis with a molecular mass coincident to SBL subunit (Figure 1(b)). In nondenaturing gels the purified protein migrated similarly as a commercially obtained SBL (Sigma Chemical Co.), although freshly obtained SBL gave a more defined band (Figure 1(a)). Furthermore, this protein had hemagglutinating activity with both group A+ human erythrocytes and *B. japonicum* USDA 110 cells, and reacted with an anti-SBL antibody (not shown). Based on these results and a previous more extensive analysis carried out with the same materials and methodology [21] we considered this preparation as purified SBL. When this protein was incubated with resting *B. japonicum* USDA 110 cells in Fåhraeus solution for 12 hours as previously described [21], it strongly bound to the bacterial surfaces, since it coprecipitated with bacterial cells after centrifugation at 10000 × g and cell lysis. To partially remove the lectin from bacterial cell surfaces, it was necessary to subject them to repeated washes in PBS containing 1 M galactose (Figure 1(c)). To confirm that such an SBL binding occurs on the EPS galactose residue we repeated the incubation with the ΔP22 *exo*B mutant strain, which produces an EPS devoid of galactose [32, 33]. In Figure 1(d) we observed that SBL was absent in protein extracts from this mutant strain, by difference with USDA 110.

### 3.2. Participation of SBL in Biofilm Formation

**Figure 2:**
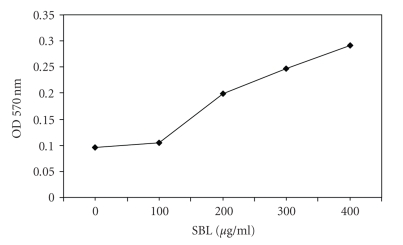
Optical density at 570 nm indicating biofilm formation in the microtiter plate assay by *B. japonicum* USDA 110 in the presence of the indicated concentrations of purified SBL, after 24-hours incubation.

**Figure 3:**
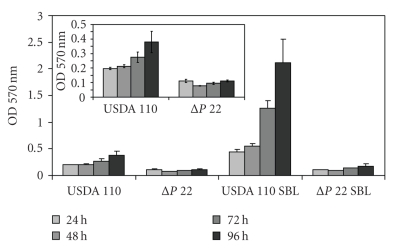
Optical density at 570 nm indicating biofilm formation in the microtiter plate assay by *B. japonicum* wild type strain USDA 110 or the EPS-defective derivative mutant ΔP22 with or without 300*μ*g mL^−1^ SBL at the indicated incubation times. In the *inset* the treatments without SBL are shown with a different scale in the ordinate axis for a better appreciation of the differences between USDA 110 and ΔP22.

 The microtiter plate assay developed by Fujishige et al*.* [10] was employed. In a preliminary characterization we observed that the wild type USDA 110 strain formed growing biofilms within a one-week period while the EPS-defective derivative ΔP22 was very inefficient. When SBL was added at the beginning of these incubations, the concentration previously used by us to increase *B. japonicum* symbiotic performance [21] was not enough to modify biofilm-forming activity. Thus, we tested a range of higher SBL concentrations, and found that with more than 100 *μ*g mL^−1^ there was a noticeable biofilm formation in repeated experiments (Figure 2). When the same treatments were applied to ΔP22, no difference between SBL-treated and controls was observed (Figure 3).

**Figure 4:**
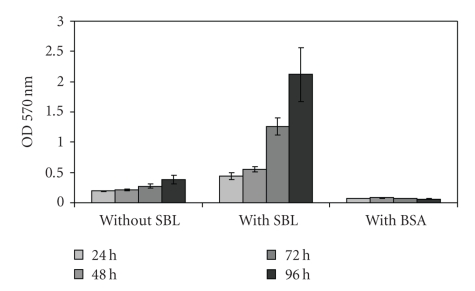
Optical density at 570 nm indicating biofilm formation in the microtiter plate assay by *B. japonicum* USDA 110 with or without 300 *μ*g ml^−1^ SBL or BSA at the indicated incubation times.

**Figure 5:**
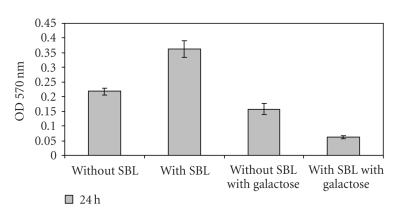
Optical density at 570 nm indicating biofilm formation in the microtiter plate assay by *B. japonicum* USDA 110 with or without 300 *μ*g ml^−1^ SBL and/or 100 mM galactose at 24-hours incubation time.

From these data we decided to continue our experiments with SBL at a concentration of 300 *μ*g mL^−1^, which gave significant differences in 24–48 hours incubations. To confirm that the biofilms were indeed enhanced by SBL and not by any protein, we replaced SBL by BSA in the same concentration and found that this protein, instead of enhancing biofilm formation, precluded it (Figure 4). The same happened with the addition of 100 mM galactose in addition to SBL to biofilms incubation; however, the same concentration of galactose by itself has much less effect on biofilm formation by USDA 110 (Figure 5). 

**Figure 6:**
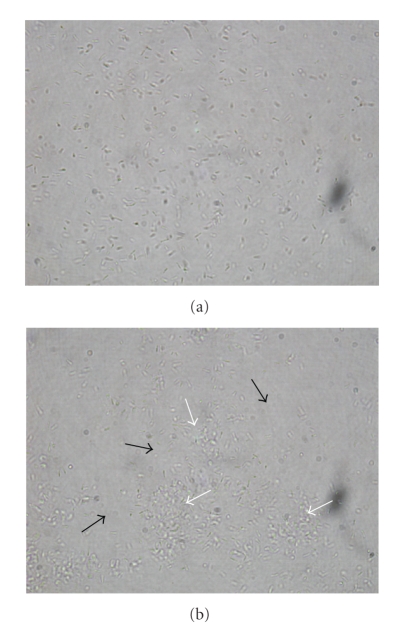
Light microscopy (1000× magnification) of *B. japonicum* USDA 110 cells obtained by careful pipetting from wells where biofilms were established by 5 days without (a) or with (b) SBL. In (b), black arrows point to spaces devoid of bacteria, and white arrows point to bacterial aggregates.

To observe the cell aggregation caused by SBL, we carefully pipetted samples from the wells of the microtiter plates five days after inoculation and observed them in the light microscope. This is only an approximate procedure since biofilms may be altered by removing cells in this way. Nevertheless, the agglutination state of SBL-treated cells could be assessed. While cells taken from wells without SBL readily dispersed producing fairly homogeneous fields (Figure 6(a)), cells taken from SBL-treated wells remained aggregated, leaving spaces without bacteria among the aggregates, thus indicating their strengthness (Figure 6(b)).

### 3.3. Adhesion of B. japonicum to Soybean Roots

**Figure 7:**
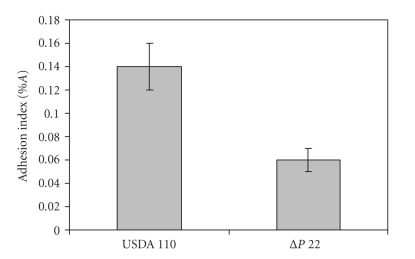
Adhesion of *B. japonicum* USDA 110 or ΔP22 to soybean roots, as quantified with the adhesion index, %A.

 The methodology employed in this work allows quantification of firm adhesion of rhizobia to plant roots, since the standardized washing procedure removes loosely bound bacterial cells [34]. In previous studies, we observed that the addition of N-acetyl D-galactosamine, a specific hapten of SBL, to a rhizobia-plant incubation medium during adhesion assays had little effect on the level of rhizobial adhesion in 4-hour incubations [20]. However, preincubation of rhizobia in SBL for longer periods increased adhesion [21], and this effect was enhanced by culturing the rhizobial cells under N-starvation, a condition that also stimulated EPS production [35]. Here we compared the adhesion of *B. japonicum* wild type USDA 110 and the derived ΔP22 strain, which is unable to incorporate galactose moieties into its EPS [32, 33] and therefore, does not bind SBL (Figure 1(d)). As shown in Figure 7, adhesion of ΔP22 to soybean roots was significantly lower than USDA 110, indicating that complete EPS and/or SBL binding are required for firm adhesion of *B. japonicum* to soybean roots.

### 3.4. SBL Stability

 We assessed SBL stability by incubating it for different periods ranging from 1 day to 1 week at 37°C or 60°C in double-distilled water in screw-capped polystyrene 1.5 mL vials. After these incubations were completed, double-distilled water was added if volume losses were detected, and the samples were cooled down to room temperature. Then, we performed serial dilutions in double-distilled water in microtiter plates, and added one volume of either human A+ erythrocytes or *B. japonicum* cells suspended in 2× PBS to measure SBL agglutinating ability. We observed that SBL agglutinating activity was 100% stable with both cell types after the complete range of temperature and incubation periods tested, even after one week at 60°C, the maximal stringency employed in our assays.

## 4. Discussion

In this work the influence of SBL in biofilm formation by *B. japonicum* in the absence of soybean plants was demonstrated. The presence of this plant lectin increased biofilm formation in amounts that depended on SBL concentration, it could not be replaced by BSA, and the presence of galactose, a known SBL hapten, was strongly inhibitory. In addition, EPS appeared as a primary factor required for biofilm formation, since the EPS-defective strain ΔP22, which produces a short EPS without galactose [32, 33, 36], did not bind SBL, had a reduced ability to form biofilms, did not respond to SBL, and did not agglutinate. In addition to its reduced ability to form biofilms in microtiter plates, ΔP22 was also impaired for root adhesion. However, the effect of SBL on root adhesion was questioned by experiments where the addition of SBL to the plant-incubation medium had no influence [21], and the presence of the potent SBL hapten N-acetyl D-galactosamine had no effects on adhesion even at 10 mM concentration [20, 37, 38]. Meanwhile, the same hapten employed in micromolar concentrations inhibited both erythrocyte agglutination [39] and adhesion stimulation by rhizobia preincubation in SBL or protein seed extracts [21], which in this case seems to act by inducing a physiological change in the rhizobia rather than by a direct action on rhizobial binding [21, 40]. Thus, reduced root adhesion of ΔP22 might be explained by a more general effect of EPS amounts and size rather than by a direct effect of SBL. By contrast to root adhesion, the presence of galactose in the incubations of microtiter biofilm assays was inhibitory. Interestingly, biofilm formation was significantly reduced in the presence of both SBL and galactose in relation to the control without any addition. This indicates that biofilm formation follows diverse pathways in the presence or absence of SBL, but we cannot anticipate whether these could be reflected in biofilm structures and properties.

The above results indicate that there exist different contributions of EPS and SBL to biofilm formation on a hydrophobic inert surface or on initial *B. japonicum* adhesion to soybean roots. While EPS affects both processes, SBL might have a direct effect on the first but not on the second. Biofilm formation is a slow process in comparison to the root infection carried out by rhizobia. It was estimated that a given soybean root hair remains infectable for only 6 hours [41] while biofilm formation and maturation typically occur in several days [5], unless highly concentrated inocula, almost two orders of magnitude above the rhizobial concentration normally encountered in soils, are employed. In this way, measures performed in soil indicate that growing roots passing at the vicinity of a rhizobial microcolony might maturate and become uninfectable faster than the time required for members of the microcolony to migrate to a developing root hair, attach to it, and develop a mature biofilm [42]. Root hair tips colonization was described as a two-step process, the first one being the adhesion of individual rhizobial cells and the second, the growth and maturation of a rhizobial cap on the root hair tip, which takes almost one day to complete. Although the rhizobia probably start to change physiologically as soon as they attach to the surface, the physiological pathway to biofilm development and maturation is not necessarily linked in any way to infection and nodulation. Beyond the different requirements of SBL for each process described above, the roles of different EPSs in *R. leguminosarum* bv *viceae* also diverge: the roles of glucomannan and cellulose were different depending on whether the biofilm was developed on glass surface or on root hairs, being these polysaccharides required only for cell adhesion and biofilm maturation on root hairs [12]. A similar behavior was observed with the RapA1 adhesin, which although is required for root colonization, seems not for adhesion or biofilm maturation on glass or plastic surfaces [43]. Moreover, biofilm formation was described in *Sinorhizobium meliloti*, which in addition to EPS required the common *nod* genes [13] and was sensitive to diverse environmental conditions, such as osmotic stress, pH, temperature, and N-nutrition [14]. The function of common *nod* genes is remarkable, since specific *nod* genes are not required, being only the core structure of the Nod factor which seems to play a role while decorations typically required for root hair infection are dispensable for biofilm formation [13]. The authors suggested that the function of Nod factor in biofilm formation might constitute an ancestral one, not directly related to symbiosis.

In this context, it may be surprising that a plant protein, also demonstrated as playing some role on host symbiotic specific recognition by the bacteria [19] may act in stimulating biofilm formation in the absence of plants, as observed in this work. Looking for an explanation of such phenomenon we measured the stability of SBL cell agglutinating activity. To this end, we subjected the purified protein to a high temperature treatment and found that even after one week at 60°C this activity was fully retained either with *B. japonicum* cells or human group A+ erythrocytes. Glycoproteins like SBL are known to be stable, and a high Δ*G* of unfolding was observed for this protein due to its strong hydrophobic core and subunit-subunit associations with participation of both glycans and aminoacid side chains [44]. Taken together, these results suggest that plant remnants in soil during soybean cropping or even after soybean harvest might be an excellent source of this stable protein, which could remain active for long periods providing nucleation sites to favor biofilm formation by *B. japonicum*. Since biofilms are widely recognized as resistant structures against factors such as desiccation, predation, antibiosis, or UV irradiance, all of which occur in naked soil after crop harvest, released SBL during soybean life cycle or even after plant death may contribute to create protecting niches for *B. japonicum* survival in the site where soybean proliferates, to better hold the interval from one soybean generation to the next.
